# Multidimensional Proteomics Reveals the Pro-apoptotic Mechanism of Platycodin D: Targeting RFC4 to Regulate the Notch Signaling Axis in Non-Small Cell Lung Cancer

**DOI:** 10.1016/j.mcpro.2026.101587

**Published:** 2026-05-16

**Authors:** Hongyu Zhang, Baojin Zhou, Jingwen Liu, Yan Ren

**Affiliations:** 1 Experiment Center for Science and Technology, Shanghai University of Traditional Chinese Medicine, Shanghai, P.R. China; 2 Institute of Basic Medicine and Cancer (HIM), Chinese Academy of Sciences, Hangzhou, Zhejiang, China

**Keywords:** Platycodin D, target proteins, RFC4, Notch, apoptosis

## Abstract

Platycodin D (PD), a major bioactive saponin isolated from the traditional Chinese medicine *Platycodon grandiflorus*, has shown promising therapeutic potential against non-small cell lung cancer (NSCLC). However, the functional mechanisms of PD in NSCLC progression remains unclear. This study aimed to explore the pharmacological mechanism of PD against NSCLC. Thermal proteome profiling approach, molecular docking, cellular thermal shift assay and peptide-centric local stability assay were employed to identify the potential binding target of PD. Subsequent Western Blot and immunoprecipitation-Western Blot experiments were conducted to investigate the downstream signaling pathways of the target. Furthermore, proteomic and ubiquitinomic profiling of PD-treated cells were performed to investigate its functions on global. replication factor C subunit 4 (RFC4) was identified as a potential binding target of PD by thermal proteome profiling and their binding sites were further exposed by peptide-centric local stability assay. PD-RFC4 complex promotes the degradation of Notch1 and Notch3 by reducing nuclear entry of their domains. Compared with control treatment, the differentially expressed proteins induced by PD were found to be primarily involved in ferroptosis, ubiquitination, platinum drug resistance, and ribosome-related processes. The ubiquitin proteome analysis revealed that proteins associated with the Notch pathway underwent ubiquitin modifications. PD binds to RFC4 and inhibits its activity, leading to downregulation of the Notch signaling pathway, ultimately triggering cancer cell apoptosis. PD is a natural product with potential therapeutic value for NSCLC.

## Highlights

• RFC4 is identified as a potential target of PD by TPP assay, CETSA and PELSA.
• PD exerts inhibitory effects on NSCLC by inhibiting RFC4/Notch signaling pathway.
• PD promotes protein ubiquitination in Notch pathway.

## Graphical Abstract

**Figure:**
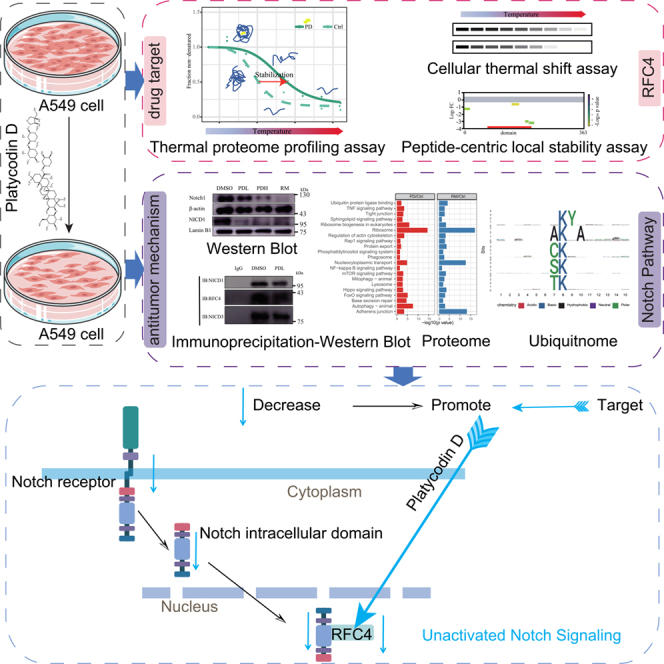

## In Brief

Zhang *et al*. employed multidimensional proteomic approaches, including thermal proteome profiling, peptide-centric local stability assay, proteome, and ubiquitinome, to demonstrate that PD exerts therapeutic effects against NSCLC by inhibiting the RFC4/Notch signaling pathway. The mechanism involved PD targeting RFC4, which suppresses the expression of Notch1 and Notch3, and reduces the nuclear translocation of NICD1 and NICD3. These findings elucidated the pharmacological mechanism of PD in NSCLC, providing a novel strategic direction for the treatment of NSCLC.

Lung cancer exhibits the highest incidence and mortality rates among all malignant tumors. Non-small cell lung cancer (NSCLC) is the most prevalent type, accounting for over 80% of global lung cancer cases (1, 2). The majority of patients are diagnosed at an advanced stage, with a 5-years survival rate of less than 15% (3), posing a severe threat to human health. Challenges in NSCLC treatment include chemotherapy-related adverse effects, complex drug resistance mechanisms, and the urgent need to optimize perioperative evaluation systems. Consequently, the discovery of novel therapeutic targets and mechanisms is crucial for developing more effective NSCLC treatments.

As a cornerstone herb for pulmonary disorders in Traditional Chinese Medicine, *Platycodon grandiflorus* possesses four primary actions: dispelling lung pathogens, resolving phlegm, draining pus, and soothing the throat. The enduring history of clinical practice stands as robust testimony to its established efficacy and safety profile, cementing its status as an invaluable component of the traditional Chinese medicine pharmacopeia. Platycodin D (PD), a triterpenoid saponin monomer isolated from the herb *P. grandiflorus*, has demonstrated inhibitory effects on cancer by interfering with multiple mechanisms in preclinical studies. Studies have shown that PD can exert antiproliferative effects, induce cell cycle arrest, promote apoptosis and autophagy, suppress metastasis, overcome drug resistance and modulate immune responses in various types of cancer including NSCLC (4, 5, 6, 7, 8). Although PD exhibits multitarget antitumor activity, researches on its specific therapeutic targets and precise mechanisms against NSCLC remains limited and warrants further exploration.

Replication factor C subunit 4 (RFC4) shows coordinate expression in a majority of tumor types, including NSCLC. RFC4 is a clamp loader protein that binds in a complex with proliferating cell nuclear antigen to regulate elongation of primed DNA templates by DNA polymerases (9, 10). RFC4 is crucial for initiating DNA replication *via* DNA polymerase δ and ε (11, 12), which is critical for cancer cell survival. Activation of the Notch signaling pathway is initiated by receptor–ligand interactions between neighboring cells. This process involves the action of 3 different proteases and γ-secretase, which mediate the nuclear translocation of the Notch intracellular domain (NICD), ultimately regulating the expression of downstream target genes (13, 14, 15). Termination of this signaling pathway is mediated through proteasomal degradation of NICD (16, 17, 18, 19). Mutations in the Notch genes are associated with the development of various cancers, including lung squamous cell carcinoma (20), breast cancer (21), and anaplastic large cell lymphoma (22). Prior study (23) showed that Notch signaling drives the stemness and tumorigenicity of NSCLC. Recent study (24) has demonstrated that RFC4 and NICD1 form a positive feedback loop that critically promotes NSCLC progression. Therefore, targeting RFC4 to suppress NICD1 could represent a potential therapeutic strategy for NSCLC.

This study aims to investigate the therapeutic potential of PD on NSCLC progression and elucidate its underlying molecular mechanisms. We conducted experiments to assess apoptosis and proliferation in lung cancer cells treated with PD. Thermal proteome profiling (TPP), cellular thermal shift assay (CETSA), peptide-centric local stability assay (PELSA) and molecular docking were employed to identify potential targets of PD. For the first time, RFC4 was discovered as a potential target of PD. By inhibiting RFC4, PD modulates the Notch signaling pathway, which reduces the nuclear import of NICD1 and NICD3 and consequently induces apoptosis in NSCLC cells. Furthermore, proteomic and ubiquitinomic experiments were conducted to gain an in-depth and comprehensive understanding of PD in NSCLC. The results demonstrated that PD exerts its effects *via* multiple pathways, such as ferroptosis, platinum drug resistance, TNF signaling pathway and ubiquitin mediated proteolysis. In addition, ubiquitinomics data suggest that PD inhibits the Notch signaling pathway by enhancing the ubiquitination of proteins related to this pathway. This finding not only provides a novel mechanistic explanation for the antitumor effects of PD but also offers a theoretical foundation for targeting RFC4 as a therapeutic strategy in NSCLC treatment.

## Experimental Procedures

### Experimental Design and Statistical Rationale

In this study, multiple proteomic techniques were employed to systematically analyze target protein identification as well as changes in protein abundance, turnover, and ubiquitination following PD treatment. We focused on the identification of target proteins with potential binding of PD as the first step to investigate its functions. TPP CETSA, and PELSA were adopted for target protein identification. Proteomic analysis of PD-treated cells was performed to investigate its global effects. The ubiquitin proteome was used to evaluate ubiquitination levels and sites of pathway-related proteins. For ubiquitinome analysis, tryptic peptides were subjected to K-ε-GG peptide enrichment to enhance the abundance and improve the identification depth of ubiquitinated peptides.

All experiments were performed with independent biological replicates, with specific n values provided in the corresponding figure legends. Comparison between multiple groups of data conforming to a normal distribution with homogeneous variances were conducted by one-way ANOVA, and all values were presented as mean ± SD. Statistical significance was set at *p* < 0.05. Further details on experimental procedures and data analysis pipelines are provided below.

### Cell Lines and Cell Culture

The NSCLC cell line A549 from the National Infrastructure of Cell Line Resource was cultured in Dulbecco's modified eagle medium, tested for *mycoplasma* contamination prior to the experiments, and maintained at 37 °C in a humidified incubator with 5% CO_2_.

### Cell Viability Assay

A549 and H1299 cells were plated into 96-well plates at a density of 5 × 10^3^ cells/well and allowed to adhere overnight. After 12, 24, 48, and 72 h of treatment with either the dimethyl sulfoxide (DMSO) or PD (#B20643, OriLeaf, purity ≥98%, CAS No. 58,479–68–8, MW: 1225.32 Da), 20 μl of MTT solution (#MB4698-1, Meilunbio) was added, and the plates were incubated at 37 °C for 4 h. Absorbance at 550 nm and 630 nm were measured using a microplate reader (Bio-tek, Synergy HT) to determine cell viability.

### Flow Cytometry

Cells were plated into 6-well plates at a density of 5 × 10^5^ cells/well and allowed to adhere overnight. After 48 h of treatment with test compounds, adherent cells were harvested. Apoptosis analysis was performed using the Annexin V-FITC/PI Apoptosis Detection Kit (#MA0220, Meilunbio) according to the manufacturer's instructions. Cell cycle analysis was performed using the Meilun Cell Cycle and Apoptosis Analysis Kit (#MA0334, Meilunbio) according to the manufacturer's instructions. Samples were then analyzed using a Cytoflex S flow cytometer (Beckman Coulter).

### TPP Assay

The TPP experiment was performed as previously reported with minor modifications (25, 26). A549 cells were treated with either PD (15 μM) or DMSO for 1 h. The cells were cultured in 150 mm dishes, washed three times with ice PBS, and then scraped gently in 800 μl of PBS containing 1% (v/v) protease and phosphatase inhibitor (#P1045, Beyotime) cocktail. The cell suspension was transferred to a 1.5 ml tube and homogenized by sonication (40 s total, 30% amplitude, 5 s on /5 s off cycles). The supernatant protein concentration was measured with the BCA kit (#P0010S, Beyotime,) and adjusted to 1 mg/ml. Each group of solutions was placed separately into 8 × 1.5 ml tubes (50 μl per tube). To normalize TPP data using indexed retention time (iRT) standard peptides (27), iRT standards were spiked in at 10 ng per sample with a standard-to-protein ratio of 1:5000 (w/w). The tubes were heated in a Digital Dry Bath (Thermo Fisher Scientific) at temperature gradient (37, 42, 47, 52, 57, 62, 67, and 72 °C) for 3 min and kept at RT for the same duration, then centrifuged at 20,000*g* for 10 min. Samples were denatured in 8 M urea and reduced with 10 mmol/L dithiothreitol for 1 h at 56 °C. Alkylation was then performed using 30 mmol/l chloroacetamide for 30 min at RT in the dark. The reaction mixture was diluted seven-fold with 500 mM ammonium bicarbonate to achieve a final concentration of 50 mM prior to digestion. All samples were digested overnight at 37 °C with a trypsin-to-substrate ratio of 1:50 (w/w) and shaken at 150 *g*. After digestion, peptides were acidified with TFA to a final concentration of 1% (pH ≤ 3.0). The peptides was then transferred to MonoSpin C18 (GL Sciences) for desalting, and the elutes were dried to completeness.

All peptide samples were dried by vacuum centrifugation and resuspended in 0.1% formic acid (FA). The peptides were then separated by nanoLC-MS/MS using an Easy nLC 1200 and analyzed by an Orbitrap Eclipse (Thermo Fisher Scientific). The samples were analyzed using a single-column system, with the column housed in a 60 °C column oven. The mobile phases consisted of 0.1% FA in water (mobile phase A) and 80% acetonitrile with 0.1% FA (mobile phase B). Separation was performed on a C18 column (25 cm × 75 μm, 1.8 μm, in-house made) at a flow rate of 250 nl/min with the following effective gradient: 0 to 3 min, 5% to 10% B; 3 to 43 min, 10% to 30% B; 43 to 53 min, 30% to 40% B; 53 to 63 min, 40% to 100% B; 63 to 70 min, 100% B. The nanoflow liquid chromatography system was directly coupled to a mass spectrometer. Peptides eluted from the LC column were ionized *via* a nanoelectrospray ionization source and analyzed using an Orbitrap Eclipse mass spectrometer. Key MS parameters were set as follows: ion source voltage, 2100 V; full MS scan range, 400 to 1600 m/z; resolution, 120,000; AGC target, Standard; and maximum ion injection time, Auto. MS/MS scans started at 110 m/z with a resolution of 50,000. The AGC target for MS/MS was set to Custom with a Normalized AGC Target of 250%, and the maximum injection time was Auto. Precursor ions selected for MS/MS fragmentation had charge states of 2^+^ to 7^+^, a minimum intensity threshold of 25,000, and were acquired in a data-dependent mode with a cycle time of 3 s between master scans. HCD fragmentation was applied with 38% normalized collision energy, and fragment ions were detected in the Orbitrap. The isolation window was set to 0.7 m/z, and dynamic exclusion was set to 60 s.

The MS raw data were searched against the SwissProt Homo Sapiens reference database (20, 481 sequences, download at 2019.01.18) using Maxquant (version 2.5.1.0) with default parameters. Briefly, the mass tolerance for precursor ion was 10 ppm and for product ion was 0.02 Da. Carbamidomethylation was specified as a fixed modification. Oxidation of methionine, acetylation of the N terminus were set as variable modifications. A maximum of 2 miscleavage sites were allowed. Identified proteins contained at least 1 unique peptide with a false discovery rate of less than 1%. The Match Between Runs (MBR) algorithm was used for increase protein identification. The LFQ intensity of each protein was normalized using iRT intensity according to the previous study (27). Protein melting curve analysis was performed using the TPP package (v3.28.0).

### CETSA-Western Blot Experiments

A549 cells were treated with either PD (15 μM) or DMSO for 1 h. Under the same protocol as described for the TPP assay, protein lysates were obtained after treatment. The resulting suspension was divided into eight tubes and heated for 3 min at temperatures ranging from 37 °C to 72 °C, followed by a 3-min cooling step at RT. The supernatant was separated by SDS-PAGE electrophoresis (Bio-Rad) and incubated with anti-RFC4 (#DF7367, Affinity Biosciences) and anti-β-actin (#AC038, Abclonal) antibodies. The bands obtained were quantified using ImageJ software.

### Peptide-centric Local Stability Assay (PELSA)

The assay was conducted according to the established protocol of an earlier study (28), with the following adjustments. A549 cells were treated with either PD (15 μM) or DMSO for 1 h. Cell lysates were subjected to three freeze-thaw cycles alternating between liquid nitrogen and a 37 °C water bath. Samples were adjusted to 1 mg/ml using lysis buffer. Trypsin was added to 50 μl of the sample at a 1:2 (w/w) enzyme-to-protein ratio (2.5 μg/μl stock), followed by: 10-s vortex mixing, digestion at 37 °C with 1000*g* shaking for 50 s, and termination by boiling at 100 °C for 8 min. Then, 165 μl of 8 M guanidine hydrochloride (3 × sample volume) was added to the boiled digest. Reduction and carbamidomethylation were performed as described in the TPP experiment. The samples were transferred to pre-equilibrated 10-kDa centrifugal filters. Combined filtrates were acidified with 1% TFA. Acidified peptides were desalted, eluted with 80% acetonitrile/0.1% TFA, and the elutes were dried completely.

Peptides were then detected using the same LC-MS platform as described in the TPP experiment, but operating in a data-independent acquisition mode. Briefly, precursor spectra ranged 400–1,250 *m/z* were collected at 120,000 resolution to reach an AGC target of 120,000. The maximum injection time was set to 50 m. Tandem mass spectral was collected from 50 × 17 *m/z* isolation windows at 30,000 resolution, AGC target 50,0000 and maximum injection time 54 ms.

The MS raw data were searched against the SwissProt Homo Sapiens reference database using Spectronaut (version 17). The search parameters are set as follows: mass tolerance for precursor ion was 10 ppm and for product ions was 0.02 Da. Carbamidomethylation was specified as fixed modification. Oxidation of methionine and acetylation of the N terminus were set as variable modifications. A maximum of 2 miscleavage sites were allowed. The identified proteins contained at least 1 unique M5peptide with a false discovery rate of less than 1%. The peptide-level quantitative values (PEP.Quantity) were exported from the search results, and a *t* test analysis was performed on the resulting peptide quantitative values to generate differential analysis results at the peptide level. Significant peptides were filtered with fold-change >2 and *p* value (*t* test) < 0.05. Based on the peptide-level quantitative results, local stability profiles of the candidate target proteins were generated with the ggplot2 (v2.4.0.1) package.

### Molecular Docking

The crystal structure of RFC4 (PDB ID: 8UMY) was retrieved from the RCSB Protein Data Bank. Protein preprocessing was conducted using PyMOL 2.3.0, during which all water molecules, nonrelevant chains, and heterologous ligands were removed to prepare the receptor for docking. Chemical structures of candidate compounds were acquired from the DrugBank (https://go.drugbank.com/) and PubChem (https://pubchem.ncbi.nlm.nih.gov) databases. Molecular docking was performed using AutoDock Tools 1.5.6, employing the Lamarckian Genetic Algorithm to predict binding conformations and affinities. The resulting docking complexes were visualized and analyzed using PyMOL 2.3.0 and Discovery Studio 2022.

### Public TCGA Data and CPTAC Data Processing

Gene expression of RFC4 from TCGA database were download using TCGAbiolinks (v2.38.0) package. Protein intensity of RFC4 from CPTAC database (https://portal.gdc.cancer.gov/) were download online. Statistics analysis and visualization were performed using ggpubr (v0.6.1) package.

### Western Blot

A549 cells were collected 18 h after PD, DMSO or Romidepsin (#A8173, APExBIO) treatment. For detection of NICD1 and NICD3, nuclear proteins were extracted using a nuclear protein extraction kit (#P0027, Beyotime) according to the manufacturer's instructions. For other protein analyses, cells were lysed using RIPA lysis buffer (#P0013B, Beyotime) containing 1% PMSF. All protein samples were separated by SDS-PAGE and transferred to nitrocellulose membranes (Invitrogen). After blocking for 1 h, the membrane was incubated with primary antibodies against RFC4 (#DF7367, Affinity Biosciences), Notch1 (#3608, Cell Signaling, USA), Notch3 (#5276, Cell Signaling), β-actin (#AC038, Abclonal) and Lamin B1 (#12987-1-AP, ProteinTech) at 4 °C overnight, followed by incubation with secondary antibody (#AS014, Abclonal) for 1 h. Protein bands were visualized using the SCG-W3000 PLUS chemiluminescence imaging system (Servicebio) and quantified using ImageJ software.

### IP-WB

A549 cells were collected 18 h after PD or DMSO treatment, lysed with IP lysis buffer (#MB9900, Meilunbio) containing 1% PMSF. For detection of NICD1 and NICD3, nuclear proteins were extracted using a nuclear and cytoplasmic protein extraction kit (#P0027, Beyotime) according to the manufacturer's instructions. Then anti-RFC4 antibody was added, followed by rotation overnight at 4 °C. The next day, Protein A agarose beads (#20334, Thermo Fisher Scientific) were added and incubated at 4 °C for 2 h with rotation. Subsequently, beads were washed five times with IP wash buffer (150 mM NaCl, 10 mM Hepes pH 7.4, 0.1% NP-40), followed by elution with 1 M glycine (pH 3.0) twice. The eluted proteins were heated at 95 °C for 5 min for analysis by Western Blot.

### Protein Extraction and Digestion

After 18 h of treatment with DMSO, PD or Romidepsin, cells were washed three times with PBS pre-chilled at 4 °C. Freshly prepared 4 °C lysis buffer [1% SDC, 150 mM NaCl, 50 mM Tris-HCl (pH = 8.0), 1% phosphorylase inhibitor, 1 × EDTA, 50 μM PR-619 (#P126704, Aladdin), 1% protease inhibitor] was added to each sample. As previously reported (29), after scraping, the cell lysates were heated at 95 °C for 10 min and further lysed using a sonicator. Proteins were reduced and carbamidomethylated under the same protocol as described for the TPP assay. All samples were digested overnight at 37 °C with a trypsin-to-substrate ratio of 1:50 (w/w) and shaken at 150*g*. After digestion, peptides were acidified with TFA to a final concentration of 1% (pH ≤ 3.0). The supernatant was desalted and peptide concentration was measured spectrophotometrically at A280 using NanoDrop (Thermo Fisher Scientific).

Peptides were detected with a nanoElute UPLC coupled with a timsTOF Pro2 Q-TOF mass spectrometer system (Bruker Daltonics). Briefly, peptides were separated using a C18 column (25 cm × 75 μm, 1.8 μm, in-house made) with a gradient of 60 min at a flow rate of 300 nl/min. Eluting peptides were then ionized *via* electrospray ionization (CaptiveSpray) using a capillary voltage of 1.7 kV and detected in DIA-PASEF mode 26 with an ion mobility range (1/k0) of 0.60 to 1.60 Vs/cm^2^. For tandem MS, the following parameters were used: number of PASEF MS/MS scans (10); total cycle time (1.16 s); target intensity (20,000); intensity threshold (2500); charge range (0–5); isolation width (2 m/z for m/z < 700 and 3 m/z for m/z > 700); collisional energy (20–59 eV).

The MS raw data were searched against the SwissProt Homo Sapiens reference database using Spectronaut (version 17). The search parameters are set as follows: mass tolerance for precursor ion was 10 ppm and for product ions was 0.02 Da. Carbamidomethylation was specified as fixed modification. Oxidation of methionine and acetylation of the N terminus were set as variable modifications. A maximum of 2 miscleavage sites were allowed. The identified proteins contained at least 1 unique peptide with a false discovery rate of less than 1%. Significant proteins were filtered with a fold-change >1.5 and *p* value (*t* test) < 0.05. Then we conducted hypergeometric-based enrichment analysis based on Gene Ontology and Kyoto Encyclopedia of Genes and Genomes (KEGG) databases.

### Enrichment of DiGly-Modified Peptides and Detection

Approximately 5 mg of peptide mixture from each group was first dissolved in 1000 μl of NETN buffer (100 mM NaCl, 1 mM EDTA, 50 mM Tris-HCl, 0.5% Nonidet P-40, pH 8.0). A total of 75 μl K-ε-GG ubiquitin remnant motif antibody bead conjugate (#PTM-1104, PTM Biolabs) was washed three times with cold PBS, then mixed with the peptide solution and incubated at 4 °C overnight and at RT for 1 h with gentle shaking. Beads were harvested by centrifugation at 1000*g* for 1 min at 4 °C. NETN buffer was used to wash the beads for 6 times separately to remove unspecific-binding peptides, followed by three times wash with deionized water. To elute peptides, 300 μl of 0.1% TFA was added to the tube and incubated for three times at RT for 30 min with gentle shaking. The combined eluates were dried to completeness.

DiGly-modified peptides were then detected using the same LC-MS platform as described in the whole proteome experiment, but operating in data-dependent acquisition mode. Briefly, eluting peptides were directly ionized *via* electrospray ionization (CaptiveSpray) using a capillary voltage of 1.7 kV. MS1 detection range was set to 100-1700 m/z and an ion mobility range was set to 0.75 to 1.30 V s/cm^2^. For tandem MS, the top 10 ions (intensity more than 10,000) were fragmented using CID mode and detected using TOF with an accumulation time of 100 ms. Dynamic exclusion was set to 30 s.

The tandem mass spectrums were analyzed using an LFQ-ubiqutin workflow in Fragpipe (v22.0) software. Briefly, protein identification was performed using MSFragger (v4.1) against the SwissProt Homo Sapiens reference database. Mass tolerance for precursor ion and product ion was 20 ppm. Carbamidomethylation was specified as a fixed modification. DiGly modification of lysine, oxidation of methionine, and acetylation of the N terminus were set as variable modifications. A maximum of variable modification on peptide was set to 2 and a maximum of 2 miscleavage sites were allowed. Identified proteins contained at least 1 unique peptide with a false discovery rate of less than 1%. Confident ubiquityl sites were considerate with localization probability more than 0.75. Significant ubiquityl sites were filtered with a fold-change >1.5 and *p* value (*t* test) < 0.05. The ubiquitination site ratios were normalized based on proteomics data. Specifically, the ratios of significantly differential proteins were extracted and used to correct the corresponding ubiquitination ratios. The associated functional and motif analyses were performed using the Differentially ubiquitinated sites were recalculating. Motif analysis was performed using the rmotifx (v1.0) package.

### Statistical Analysis

All data in this study were analyzed using SPSS 26.0 statistical software and visualized using GraphPad Prism 9.0 software. Comparison between multiple groups of data conforming to a normal distribution with homogeneous variances were conducted by one-way ANOVA, and all values were presented as mean ± SD. Statistical significance was set at *p* < 0.05.

## Results

### PD Promoted A549 and H1299 Cells Apoptosis and Cell Cycle Arrest

**Fig. 1:**
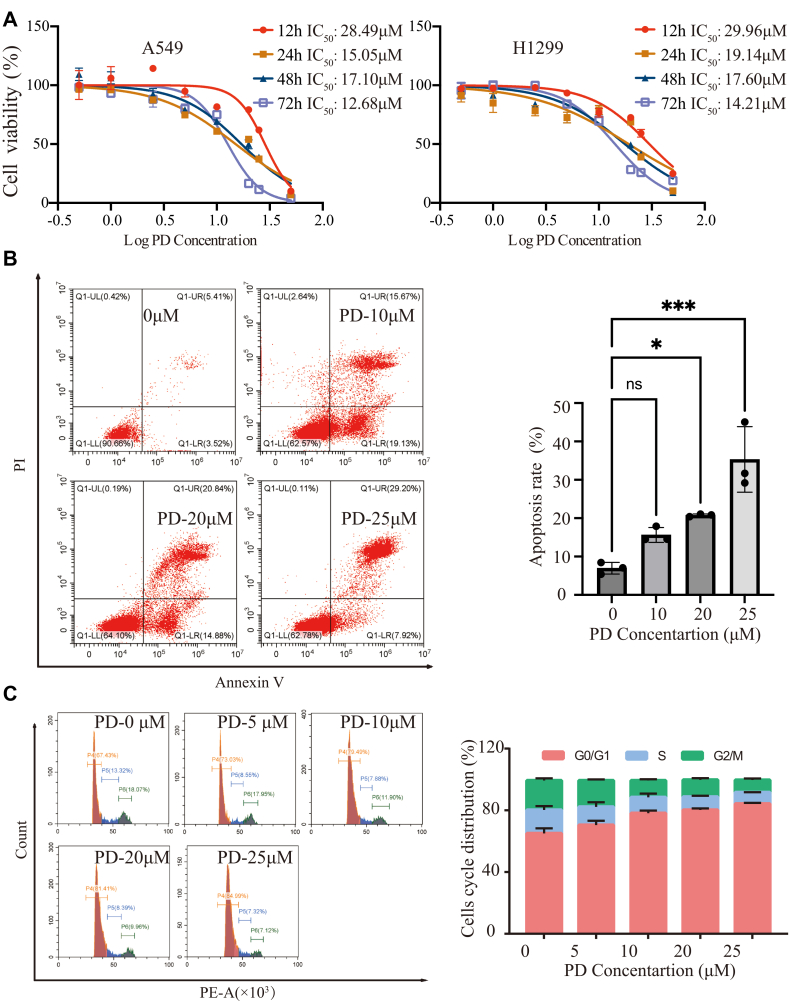
**PD impairs NSCLC cell viability by inducing late apoptosis and cell cycle arrest.***A*, cell proliferation in A549 and H1299 cells was determined by MTT assay (n = 6). *B*, detection of apoptosis levels in A549 cells by flow cytometry (n = 3). *C*, detection of Cell Cycle in A549 Cells by flow cytometry (n = 6). Data are shown as the mean ± SD. ∗*p* < 0.05, ∗∗*p* < 0.01, and ∗∗∗*p* < 0.001 *versus* untreated control cells. PD, Platycodin D.

To determine the cytotoxic effect of PD in NSCLC, human A549 and H1299 cells were treated with PD for 12, 24, 48, and 72 h (Fig. 1*A*). For A549 and H1299 cells, PD reduced cell viability in a time- and dose-dependent manner, with the most significant changes observed at 10 μM (12 h), 2.5 μM (24 h), 2.5 μM (48 h), and 2.5 μM (72 h). For A549 cells, IC_50_ values calculated from four independent experiments were 28.69 ± 2.31 μM (12h), 15.10 ± 1.46 μM (24 h), 17.17 ± 1.65 μM (48 h), and 12.67 ± 1.15 μM (72 h). For H1299 cells, IC_50_ values calculated from four independent experiments were 30.14 ± 2.20 μM (12 h), 19.67 ± 4.39 μM (24 h), 17.69 ± 2.14 μM (48 h), and 14.24 ± 0.96 μM (72 h). As determined by MTT assay, A549 cells exhibited greater sensitivity to PD treatment than H1299 cells. Accordingly, A549 cells were used in all further experiments. To detect whether the intensive proliferative inhibition was due to the enhanced induction of cell apoptosis, A549 cells were incubated with PD for 48 h, and the percentages of apoptotic cells were measured by Annexin V/PI staining assay. As shown in Figure 1*B*, PD treatment significantly increased apoptotic cell percentages from 5.41% (DMSO) to 15.67% (10 μM PD), 20.84% (20 μM PD), 29.20% (25 μM PD) in A549 cells. Changes in the cell cycle were observed after 48 h of exposure to PD at concentrations of 5 μM, 10 μM, 20 μM, and 25 μM. PD induced cell cycle arrest in the G0/G1 phase, while concurrently decreasing the S phase and G2/M phase (Fig. 1*C*). Taken together, PD inhibits the growth of NSCLC cells by inducing apoptosis and cell cycle arrest.

### RFC4 was Identified as a Potential Binding Protein of PD by TPP Assay and PELSA

**Fig. 2:**
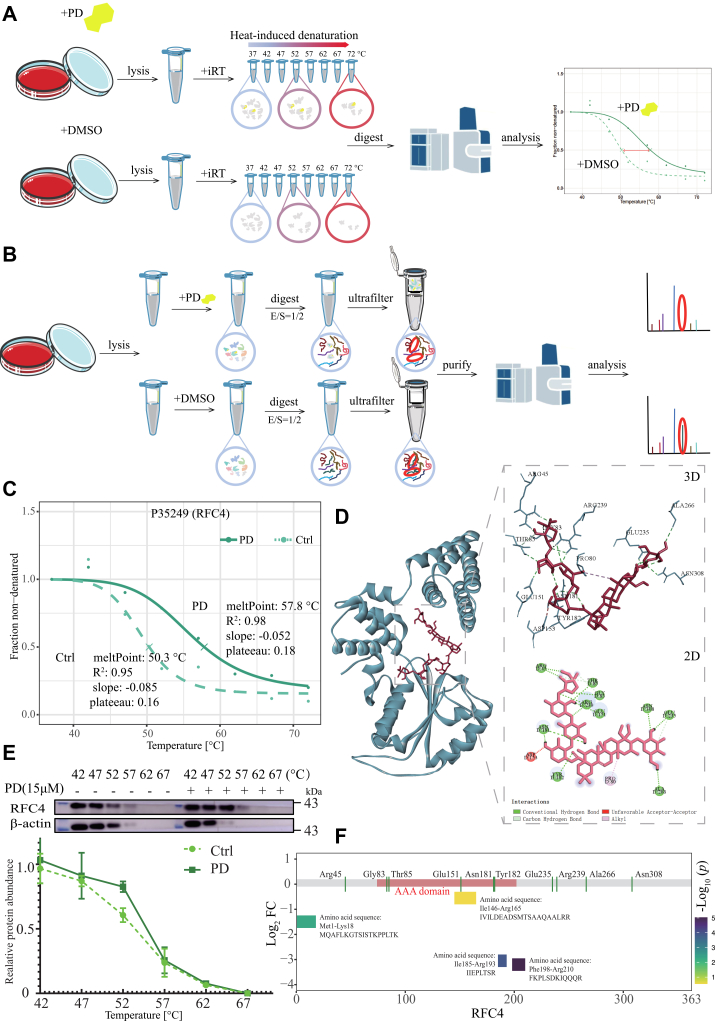
**Identification of RFC4 as atarget of PD in A549 cell.***A*, schematic of the TPP assay. *B*, schematic of the PELSA. *C*, the melting curve of RFC4 shifted significantly upon drug treatment using TPP package. *D*, docking-predicted binding pose of PD in the active sites of RFC4. *E*, representative Western blot of RFC4 following heat treatment in the presence of PD or DMSO, and quantification of the soluble RFC4 protein levels. Data are presented as mean ± SD (n = 3). *F*, local stability map of RFC4. X-axis: amino acid sequence, Y-axis: log_2_ fold change (log_2_ FC), AAA domain information is derived from the InterPro database as annotated in UniProt. PD, Platycodin D; iRT, indexed retention time; RFC4, replication factor C subunit 4; Ctrl, control.

Drug target proteins, especially those of with direct bind are important for drug effects. The TPP technology, which is an *in vivo* assay and does not require chemical modification, was adopted to identify target proteins with altered thermal stability in the presence of PD. This strategy avoids potential alterations to the native activity of PD that may arise from chemical derivatization as PD is supplemented during cell culture. The same amount of proteins were treated under an eight-temperature gradient, and the proteins remained in supernatants were digested into peptides following with reduction and alkylation, which were identified and quantified by LC-MS/MS in control and PD treatment groups, respectively (Fig. 2*A*). The thermal melting curves of proteins were generated by TPP package, which clearly showed the ΔTm and R^2^ of the same protein from control and PD treatment groups. The ΔTm values between control and PD treatment groups indicated the impacts of PD binding to targets and the R^2^ showed the trends of protein quantification change under gradient heat treatment (Table S1, Supporting Information). Proteins with R^2^ more than 0.85 and ΔTm more than 4 °C were then used to perform KEGG pathway enrichment analysis using hypergeometric test (Fig. S1, Supporting Information). Functional enrichment analysis revealed that RFC4 is involved in the first three important pathways (DNA replication, Mismatch repair, and Nucleotide excision repair). RFC4, a DNA replication factor closely associated with the cell cycle, has been reported to be amplified in NSCLC and to form a positive feedback loop with Notch signaling (24), promoting tumor metastasis and stemness. Hence, RFC4 was a potential candidate for PD's anti-NSCLC mechanism. The curve indicated the thermal stability of RFC4 was enhanced in the presence of PD (ΔTm = 7.5 °C) with a higher R^2^ (Fig. 2*C*). Molecular docking between PD and RFC4 demonstrated high-affinity binding with a favorable score of approximately −8.1 kcal/mol, which supported that PD potentially interacts with RFC4. The results suggested that PD may bind to RFC4 by accessing its pocket and establishing hydrogen bonds (Fig. 2*D*). To further confirm their binding, CETSA and PELSA assays (Fig. 2*B*) were used to visualize the binding in a sensitive way and get the binding sites of RFC4 with PD. CETSA confirmed that PD treatment increased the thermal stability of RFC4 compared to the DMSO control (Fig. 2*E*). Consistent with this, PELSA profiling also highlighted RFC4 as a key binding candidate among several putative targets (Table S2, Supporting Information). PD binding increased the regional stability of its target proteins, leading to less peptide release, which could be identified and quantified by LC-MS. A total of 5 peptides of RFC4 were quantified, in which 4 showed significant downregulation. Although only a limited number of peptides were identified for RFC4, all of them exhibited significant stability changes and were clustered within known functional domain (Fig. 2*F*). PELSA-derived peptide-level mapping suggested that PD primarily interacts with peptides located in the ATPase family associated with various cellular activities domain (AAA domain, IPR003959) of RFC4. This domain is responsible for ATP binding and hydrolysis. It plays key roles in diverse cellular processes including cell cycle regulation, protein proteolysis and disaggregation, organelle biogenesis and intracellular transport (30). The molecular docking-predicted binding sites were mapped onto the local stability profiles of RFC4 under 15 μM PD treatment. Among these, a portion of the predicted binding sites was located within the AAA domain (Fig. 2*F*). Collectively, these results consistently indicate that PD targets RFC4, thereby supporting RFC4 as a relevant biological target of PD in NSCLC cells.

### PD Inhibits the RFC4/Notch Signaling Pathway

**Fig. 3:**
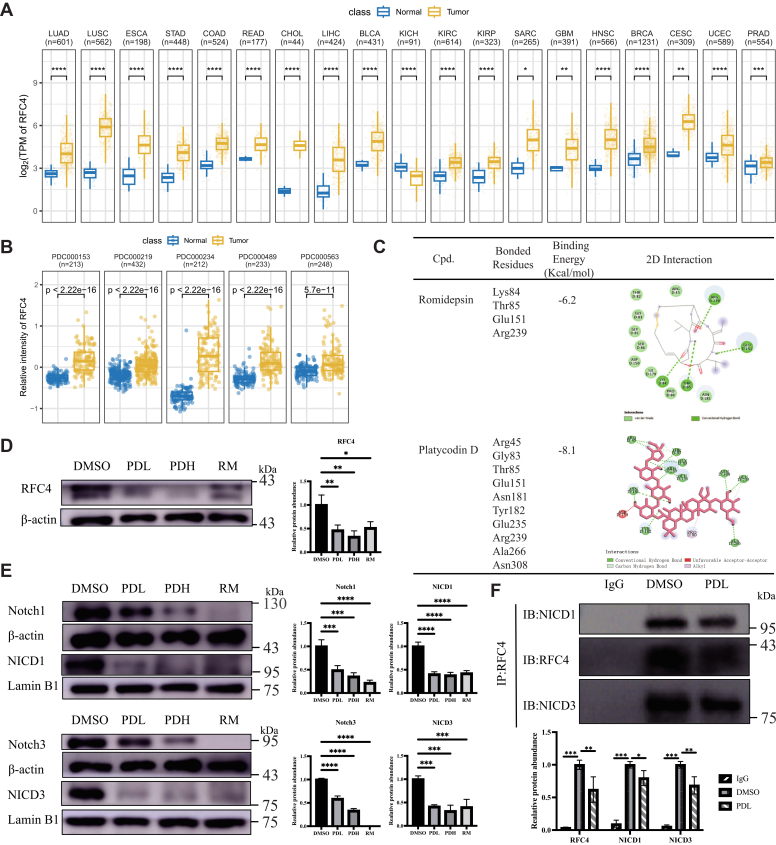
**Effects of PD and RM on RFC4 expression and Notch signaling in NSCLC.***A*, the transcripts per million of *RFC4* in different types of cancer from TCGA database. *B*, the relative intensity of RFC4 expression in NSCLC from CPTAC database. *C*, the molecular binding sites of RFC4 and Romidepsin. *D*, Western blot analysis of RFC4 and β-actin (loading control) levels in A549 cells. *E*, Western blot analysis of Notch1, NICD1, Notch3, NICD3, and β-actin or Lamin B1 (loading control) levels in A549 cells. *F*, immunoprecipitation assay revealing the interaction between RFC4 with NICD1 and NICD3. PDL, Platycodin D at a low concentration (15 μM); PDH, Platycodin D at a high concentration (25 μM); RM, Romidepsin (75 nM). Data are shown as mean ± SD (n = 3). ∗*p* < 0.05,∗∗*p* < 0.01, and ∗∗∗*p* < 0.001 *versus* untreated control cells. LUAD, lung adenocarcinoma; LUSC, lung squamous cell carcinoma; ESCA, esophageal carcinoma; STAD, stomach adenocarcinoma; COAD, colon adenocarcinoma; READ, rectum adenocarcinoma; CHOL, cholangiocarcinoma; LIHC, liver hepatocellular carcinoma; BLCA, bladder urothelial carcinoma; KICH, kidney chromophobe; KIRC, kidney renal clear cell carcinoma; KIRP, kidney renal papillary cell carcinoma; SARC, sarcoma; GBM, glioblastoma multiforme; HNSC, head and neck squamous cell carcinoma; BRCA, breast invasive carcinoma; CESC, cervical squamous cell carcinoma and endocervical adenocarcinoma; UCEC, uterine corpus endometrial carcinoma; PRAD, prostate adenocarcinoma; Cpd, compound.

Transcriptome data from TCGA database showed that *RFC4* was highly expressed in most tumor tissues. Lung adenocarcinoma and lung squamous cell carcinoma, the two major NSCLC subtypes, exhibited the most prominent overexpression (Fig. 3*A*). However, its statistical power was limited by the scarcity of normal tissue. Nevertheless, existing evidence from CPTAC database (31, 32, 33, 34) with well-matched sample sizes consistently indicated an overexpression of RFC4 in NSCLC (Fig 3*B*). The higher RFC4 expression in NSCLC might affect prognosis by promoting tumor proliferation. Recent studies (24) have revealed a critical interplay between RFC4 and Notch signaling in NSCLC. Specifically, RFC4 functions as a direct transcriptional target of Notch1 signaling, forming a positive feedback loop wherein RFC4 binds to NICD1 and competitively abrogates CDK8/FBXW7-mediated degradation of NICD1, thereby sustaining Notch signaling overactivation and promoting tumor metastasis and stemness. Given the correlation between RFC4 and Notch signaling pathway, the expression levels of RFC4 and the Notch signaling pathway in A549 cell were measured to offer global insights into the regulatory mechanism of PD. Romidepsin, an RFC4 inhibitor (35), shares same binding sites with PD (Fig. 3*C*). Treatment with PD or Romidepsin significantly inhibited the RFC4 expression level (Fig. 3*D*). Concurrently, PD treatment at 15 and 25 μmol/L markedly downregulated the expression of Notch1 and Notch3 (*p* < 0.001) and hindered the nuclear translocation of NICD1 and NICD3 to a degree comparable to Romidepsin (Fig. 3*E*). Through immunoprecipitation-Western Blot (IP-WB) assay, the endogenous interaction between RFC4 and NICD1 as well as NICD3 was successfully detected in A549 cells. Densitometric analysis revealed that, compared with the control group, treatment with 15 μmol/l PD reduced the binding of RFC4 to NICD1 by approximately 20% (*p* < 0.05) and the binding of RFC4 to NICD3 by approximately 30% (*p* < 0.05) (Fig. 3*F*). Considering that PD significantly downregulated RFC4 protein expression (Fig. 3*D*), the observed reduction in these interactions may be partially attributable to decreased total RFC4 protein levels and a potential direct interference with the RFC4-NICD interaction. Together, these data suggest that PD may interfere with Notch signaling activation by suppressing RFC4 expression and subsequently attenuating its interaction with NICD1 and NICD3.

### Proteome in A549 Cells Treated by PD or Romidepsin

**Fig. 4:**
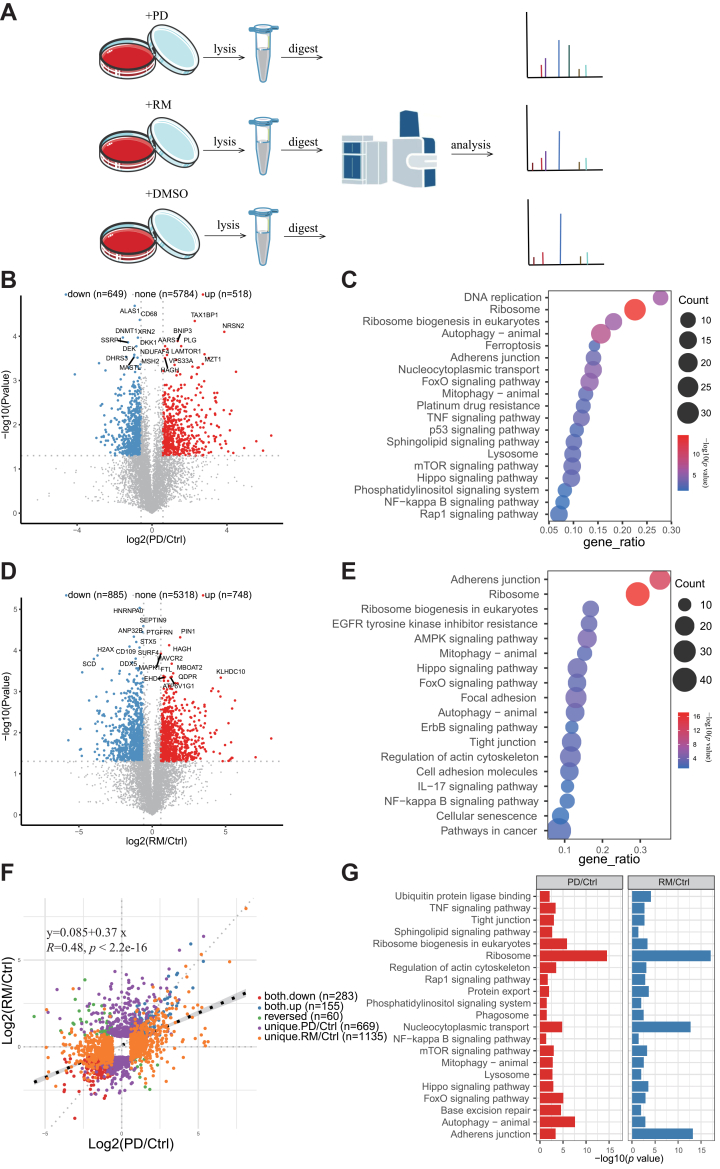
**Analysis of DEPs in A549 cells treated with PD or Romidepsin.***A*, workflow of proteomics of A549 cells treated with PD or Romidepsin. *B*, volcano plot of PD treatment *versus* control group. *C*, enriched pathways for DEPs in the PD group. *D*, volcano plot of Romidepsin treatment *versus* control group. *E*, enriched pathways for DEPs in the Romidepsin group. *F*, correlation of altered proteins across multiple comparison groups. *G*, altered pathways in both PD and Romidepsin treatments. PD, Platycodin D; RM, Romidepsin; Ctrl, control.

To further investigate the molecular mechanism of PD, a proteomics analysis on A549 cells treated with PD (18 h, 15 μM) or Romidepsin (18 h, 75 nM) was performed (Fig. 4*A*). As a result, a total of 7007 proteins were identified and quantified at 1% FDR (Table S3, Supporting Information). In the PD-treated group, a total of 1166 differentially expressed proteins (DEPs) were identified, comprising 517 upregulated and 649 downregulated proteins (Fig. 4*B* and Table S4, Supporting Information). For comparison, the Romidepsin treatment yielded 1632 DEPs, comprising 747 upregulated and 885 downregulated proteins (Fig. 4*D* and Table S5, Supporting Information). These results indicated that both treatments significantly perturbed the proteome of A549 cells. The KEGG pathway enrichment of the DEPs induced by PD were involved in tumor suppression, including the FoxO signaling pathway, TNF signaling pathway, p53 signaling pathway, Hippo signaling pathway, and NF-κB signaling pathway (Fig. 4*C*). It is noteworthy that Romidepsin also enriched some overlapping pathways (Fig. 4*E*). Interestingly, the ferroptosis pathway and Platinum drug resistance were uniquely enriched in PD-treated group, which is consistent with previous reports (36, 37). Correlation analysis revealed a positive concordance (*R* = 0.48, *p* < 2.2 × 10^−16^) between the proteomic profiles altered by PD and Romidepsin (Fig. 4*F*). Among these altered biological pathways, to identify more reliable pathways, we selected the biological pathways commonly altered by PD and Romidepsin (Fig. 4*G*). Specifically, these include ubiquitin protein ligase binding, TNF signaling pathway, Ribosome, and autophagy. These pathways suggest consistency with their shared phenotype of inducing cancer cell apoptosis.

### Ubiquitylome in A549 Cells Treated by PD

**Fig. 5:**
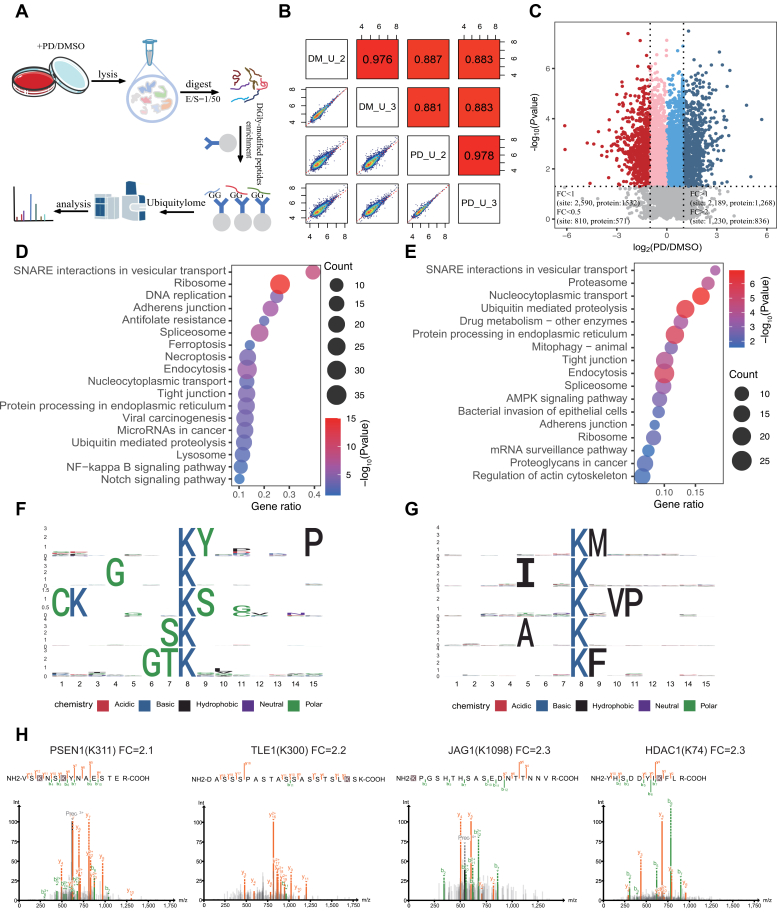
**Ubiquitylome in A549 cells before and after PD treatment.***A*, the workflow of ubiquitylome analysis of NSCLC. *B*, correlation analysis of ubiquitinomics samples. *C*, changes in protein ubiquitination sites before and after treatment. *D*, functional enrichment analysis of upregulated proteins identified through integrated proteomic and ubiquitinated proteomic analysis. *E*, functional enrichment analysis of downregulated proteins identified through integrated proteomic and ubiquitinated proteomic analysis. *F*, motifs in a subset of upregulated peptides. *G*, motifs in a subset of downregulated peptides. *H*, fragmentation spectrum of ubiquitinated peptides. PD, Platycodin D; DM_U, ubiquitylome of A549 cells treated with DMSO; PD_U, ubiquitylome of A549 cells treated with PD; FC, fold change.

As describe above, both treatments were associated with ubiquitin protein ligase binding, which regulate key behaviors of tumor cells, including proliferation, invasion, and apoptosis. Particularly in NSCLC, aberrant expression of specific ubiquitination-related proteins is closely associated with disease progression. Moreover, Termination of Notch signaling pathway is intimately associated with the ubiquitin-proteasome system (16, 17, 18, 19). Ubiquitinomic profiling was performed to systematically identify all ubiquitinated proteins following PD treatment (Fig. 5*A*). A total of 9983 ubiquitination sites and 3330 proteins were identified across the treatment and control groups (Table S6, Supporting Information). The high biological replicate correlation (r = 0.97) indicates good quantitative quality of our ubiquitinome data. The subsequent reduction in intergroup correlation to 0.8 suggested that PD induces alterations in the ubiquitinated proteome. In detail, PD treatment yielded 1230 upregulated ubiquitin sites corresponded to 836 proteins (fold change >2, *p* value < 0.05), as well as 810 downregulated sites corresponded to 571 proteins (fold change <0.5, *p* value < 0.05; Fig. 5*C*). Significant upregulated ubiquitination proteins induced by PD treatment were involved in key biological pathways, including Notch signaling pathway, ferroptosis, DNA replication, NF-κB signaling pathway and nucleocytoplasmic transport (Fig. 5*D*), consistent with our previous findings. Significant downregulated ubiquitination proteins induced by PD treatment were participated in key biological pathways, including AMPK signaling pathway, ubiquitin mediated proteolysis, endocytosis, proteasome and endocytosis (Fig. 5*E*).

**Fig. 6:**
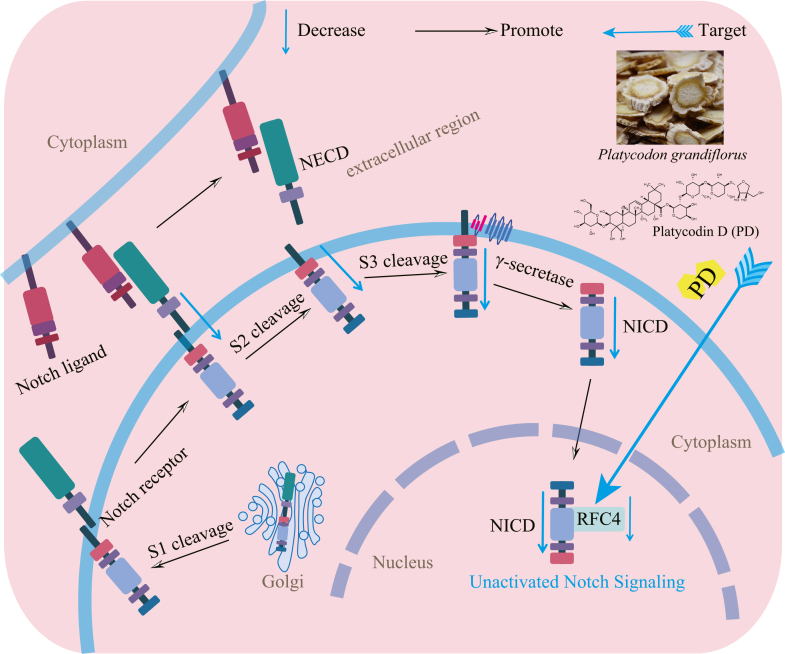
**Mechanism diagram.** Platycodin D induces apoptosis in non-small cell lung cancer cells by targeting RFC4 to regulate Notch signaling axis. NECD, notch extracellular domain; NICD, notch intracellular domain.

Our data indicate PD perturbs the ubiquitinome, affecting thousands of proteins. The global ubiquitination and subsequent degradation of these proteins are tightly regulated by complex mechanisms. Given that E3 ubiquitin ligases and deubiquitinases often target protein substrates with shared structural motifs, we performed a motif analysis on the sequences surrounding the dysregulated ubiquitination sites to investigate the underlying mechanism. Our analysis revealed distinct motif patterns between upregulated (Fig. 5*F*) and downregulated (Fig. 5*G*) sites. Upregulated motifs favor polar amino acids near the ubiquitinated lysine, while downregulated motifs prefer hydrophobic residues. These motif patterns offer new perspectives on the substrate specificity of the ubiquitination machinery in PD. Following the identification of RFC4 as a plausible target, particular attention was given to the ubiquitination of Notch pathway-related proteins, due to its known regulatory circuit with RFC4 (24). Among the ubiquitin proteins, we identified 4 upregulated proteins related to Notch signaling pathway (Fig. 5*H*), including K311 of Presenilin-1(PSEN1), K1098/K1142 of protein jagged-1, K74 of histone deacetylase 1 and K300/K316 of transducin-like enhancer protein 1. Prior studies (38, 39, 40, 41) have demonstrated the involvement of these proteins in promoting Notch signaling activation. We speculate that alterations in protein ubiquitination in the Notch signaling pathway are influenced by the PD-RFC4 complex (Fig 6).

## Discussion

This study demonstrated that PD significantly inhibits the proliferation of NSCLC cells *in vitro*. To elucidate the underlying mechanism, TPP was adopted to get the potential binding proteins of PD and identified RFC4 as a potential therapeutic target of PD for the first time. Through a series of experimental validations, we confirmed that PD binds to RFC4 and inhibits its activity, leading to downregulation of the Notch signaling pathway. Mechanistically, PD reduced the expression of Notch1 and Notch3 and impaired the nuclear translocation of their intracellular domains due to enhanced ubiquitination, ultimately triggering cancer cell apoptosis. These findings elucidate the molecular mechanism underlying PD's antiproliferative effects.

RFC4 was significantly overexpressed in NSCLC tumors compared with matched normal tissues (42). As a major subtype of NSCLC, lung adenocarcinoma exhibits high RFC4 expression, which makes it a predictive biomarker for tumorigenesis and poor prognosis, as well as a potential therapeutic target (43). As reported by previous research (44), RNAi constructs targeting RFC4 induced a commensurate decrease in anchorage-independent growth and cellular invasion of A549 and H358 cells. Romidepsin is an established RFC4 inhibitor that shares overlapping binding sites with PD. Western blot analysis demonstrated comparable phenotypic effects between high-concentration PD and romidepsin. PD displays a favorable safety profile and functions as a polypharmacological agent modulating multiple targets and pathways. In our previous work, integrated whole-cell proteomic and ubiquitination proteomics profiling identified that PD could regulate several pathways that exert multiple effects on NSCLC proliferation. To be specific, our proteomic analysis indicated that PD-induced increase in ubiquitination resulted in the blockade of several critical signaling pathways. Notably, it is well established in the literature that the inhibition of the FoxO (45, 46), p53 (47, 48), NF-κB (49, 50, 51), Hippo (51, 52), and TNF (53, 54) signaling pathways is closely linked to cancer cell apoptosis.

It is noteworthy that the ferroptosis pathway and platinum drug resistance were demonstrated to be highly significant in our results. Furthermore, previous studies have demonstrated that the administration of PD can ameliorate pathologies of various diseases, including polycystic ovary syndrome (55), prostate cancer(56). and diabetic kidney disease (57, 58) by modulating ferroptosis. Notch1 and Notch3 are important receptors involved in mediating tumor drug resistance. Studies have shown that activation of Notch1 signaling promotes tamoxifen resistance in breast cancer xenograft tumors, confers resistance of melanoma cells to MAPK inhibitors (59). Previous studies (60, 61, 62) have shown that Notch1 has been implicated in resistance to multiple drugs in NSCLC. In addition, elevated expression of Notch3 has been shown to mediate cisplatin resistance in epithelial ovarian cancer cells (63). These observations provide a strong rationale for future studies into these mechanisms.

The work of Liu *et al*. (24) established that elevated expression of RFC4 binds to NICD1 and competitively inhibits its ubiquitination by E3 ligases, thereby stabilizing NICD1 and enhancing its transcriptional activity. Since RFC4 itself is a downstream target gene of NICD1, a positive feedback loop is established, which promotes metastasis and stemness in NSCLC. Interestingly, our Western blot analysis revealed that RFC4 expression was downregulated following drug administration; ubiquitin proteomics analysis revealed PD-induced upregulation of ubiquitination at specific sites within the Notch signaling pathway. These findings demonstrated consistent changes in ubiquitination patterns that align with prior reports. In addition to its interaction with NICD1, our IP-WB data showed that RFC4 also potentially binds to NICD3.

High levels of Notch3 and NICD3 are implicated in the pathogenesis of a variety of cancers, including prostate cancer (64), ovarian cancer (65) and NSCLC. Suppression of Notch3 and NICD3 expression promotes apoptosis, reduces drug resistance, and impairs metastatic capabilities in these cancer cells. Inhibitor of Notch3 enhances the sensitivity of NSCLC cells to gemcitabine (66). It has been reported that one degradation pathway of NICD3 is mediated by the E3 ubiquitin ligase Itchy homolog *via* the mono-ubiquitin lysosomal pathway in NSCLC (67). Consistent with earlier studies, our Western blot data corroborated the decrease in NICD3 after drug administration, which led to significant apoptosis in A549 cells.

Collectively, we have integrated multiple proteomic approaches to explore the targets and pro-apoptotic mechanisms of PD in the treatment of NSCLC, demonstrating the therapeutic potential of PD as a candidate drug for NSCLC treatment. Our work not only paves the way for novel targeted therapies against NSCLC but also advances the mechanistic understanding of RFC4's biological functions. However, we acknowledge that the mechanisms underlying the degradation of RFC4 and Notch-related proteins are not fully elucidated. More studies that can reveal the direct interaction of RFC4-PD and RFC4-NICD should be employed including intracellular proximity-based labeling approaches and surface plasma resonance. Moreover, we cannot rule out that PD may engage additional targets and broader regulatory networks. Further in-depth *in vitro* and *in vivo* research is required to decipher the polypharmacology of PD and explore combination therapies to establish the foundation for clinical translation.

## Data Availability

The MS proteomics data have been deposited in the ProteomeXchange Consortium *via* the iProX partner (68) repository with the dataset identifier PXD071858 (ProteomeXchange) and IPX0014490000 (iProX).

## Supplemental Data

This article contains supplemental data.

## Conflict of Interest

The authors declare no competing interests.
