# Supplementary material for: Multidimensional Proteomics Reveals the Pro-apoptotic Mechanism of Platycodin D: Targeting RFC4 to Regulate the Notch Signaling Axis in Non-Small Cell Lung Cancer
Source: Mol Cell Proteomics. 2026 May 16;25(8):101587. doi: 10.1016/j.mcpro.2026.101587 (PMC13469972; doi:10.1016/j.mcpro.2026.101587)
Supplement: Supplementary file 1 [file mmc7.pdf]

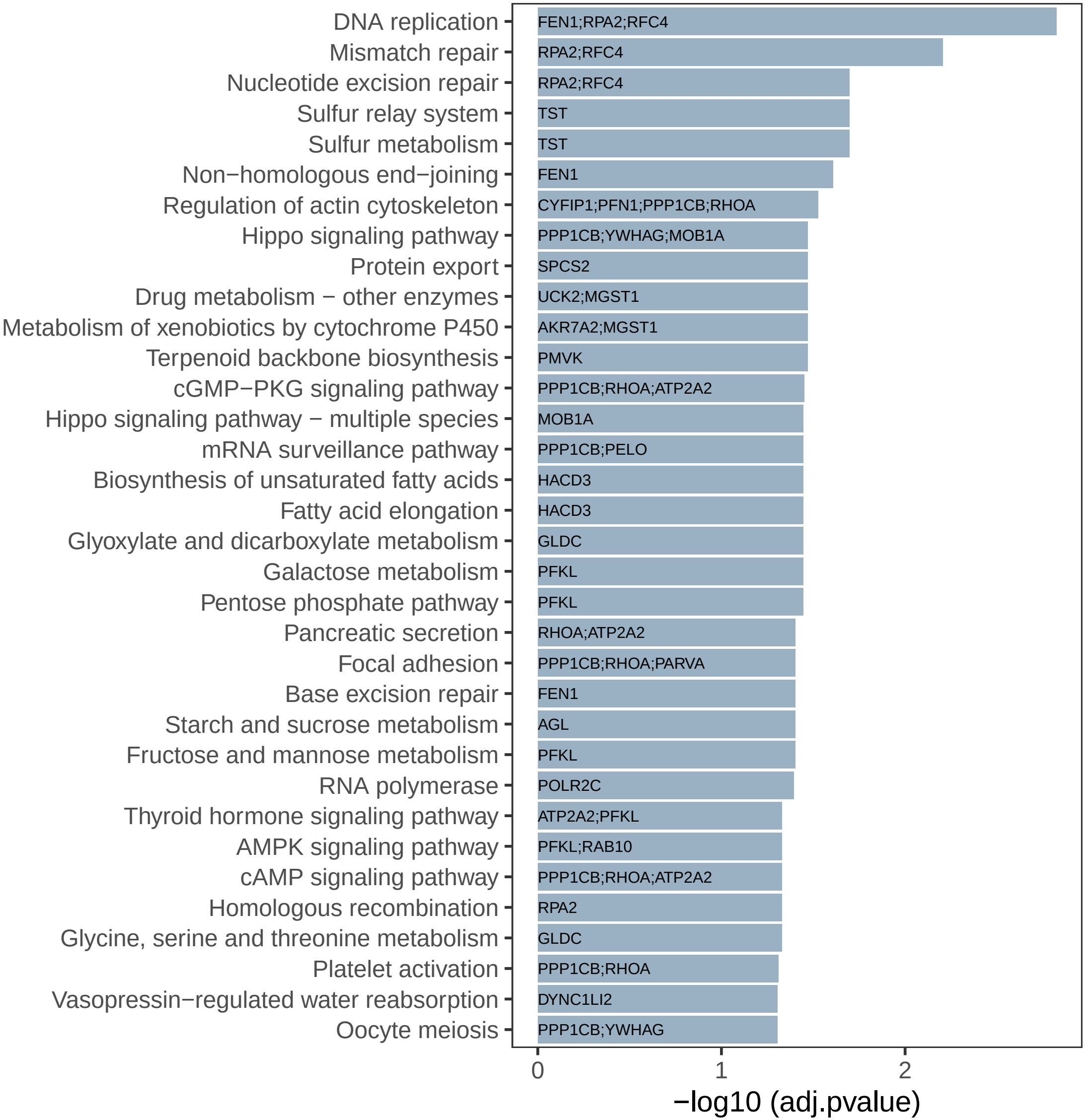
DNA replication
Mismatch repair
Nucleotide excision repair
Sulfur relay system
Sulfur metabolism
Non−homologous end−joining
Regulation of actin cytoskeleton
Hippo signaling pathway
Protein export
Drug metabolism − other enzymes
Metabolism of xenobiotics by cytochrome P450
Terpenoid backbone biosynthesis
cGMP−PKG signaling pathway
Hippo signaling pathway − multiple species
mRNA surveillance pathway
Biosynthesis of unsaturated fatty acids
Fatty acid elongation
Glyoxylate and dicarboxylate metabolism
Galactose metabolism
Pentose phosphate pathway
Pancreatic secretion
Focal adhesion
Base excision repair
Starch and sucrose metabolism
Fructose and mannose metabolism
RNA polymerase
Thyroid hormone signaling pathway
AMPK signaling pathway
cAMP signaling pathway
Homologous recombination
Glycine, serine and threonine metabolism
Platelet activation
Vasopressin−regulated water reabsorption
Oocyte meiosis
FEN1;RPA2;RFC4
RPA2;RFC4
RPA2;RFC4
TST
TST
FEN1
CYFIP1;PFN1;PPP1CB;RHOA
PPP1CB;YWHAG;MOB1A
SPCS2
UCK2;MGST1
AKR7A2;MGST1
PMVK
PPP1CB;RHOA;ATP2A2
MOB1A
PPP1CB;PELO
HACD3
HACD3
GLDC
PFKL
PFKL
RHOA;ATP2A2
PPP1CB;RHOA;PARVA
FEN1
AGL
PFKL
POLR2C
ATP2A2;PFKL
PFKL;RAB10
PPP1CB;RHOA;ATP2A2
RPA2
GLDC
PPP1CB;RHOA
DYNC1LI2
PPP1CB;YWHAG
0
1
2
−log10 (adj.pvalue)
